# Supplementary material for: Unraveling the lncRNA-miRNA-mRNA Regulatory Network Involved in Poplar Coma Development through High-Throughput Sequencing
Source: Int J Mol Sci. 2024 Jul 5;25(13):7403. doi: 10.3390/ijms25137403 (PMC11242837; doi:10.3390/ijms25137403)
Supplement: Supplementary file 1 [file ijms-25-07403-s001.zip › Supplementary figures.pdf]

Supplementary Figures

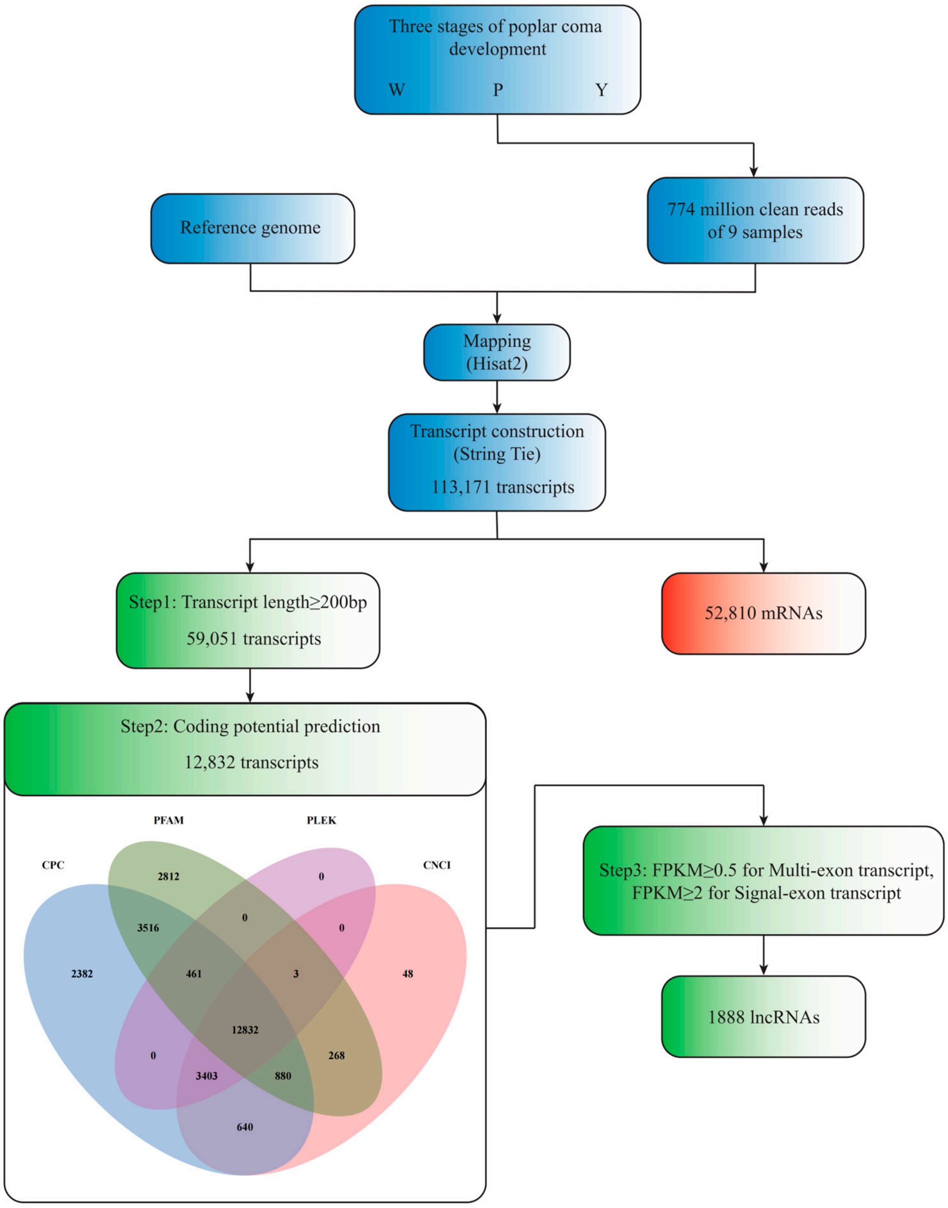

Figure S1. The flowchart for the identification of lncRNA and mRNA.

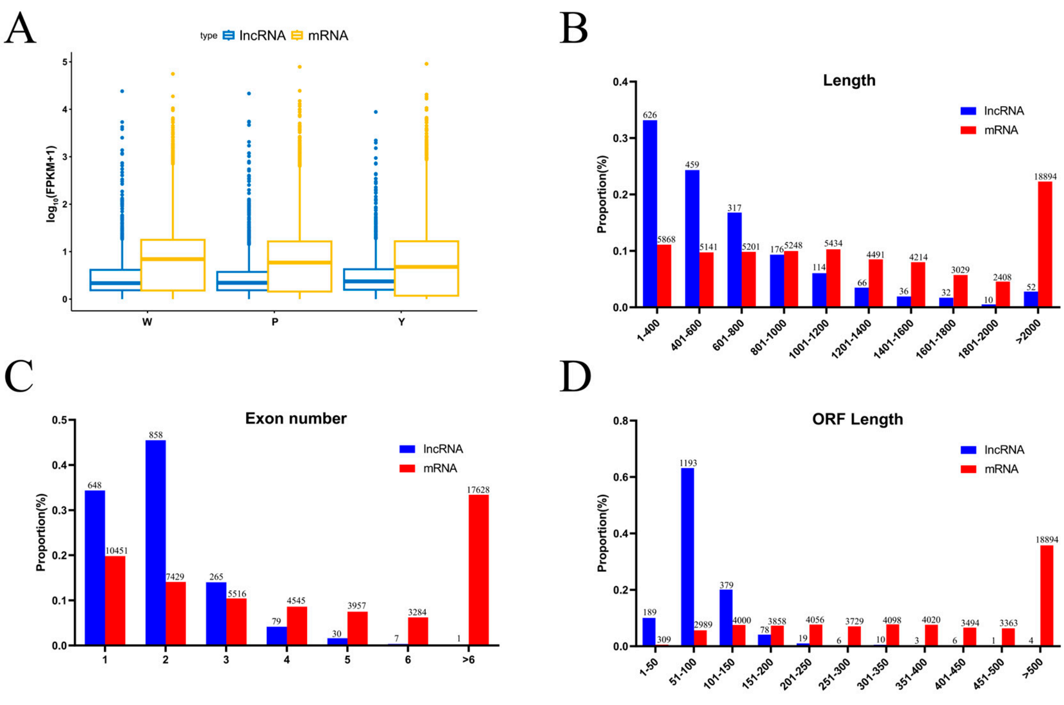

Figure S2. Comparison of features between lncRNAs and mRNAs. A. Comparison of expression levels between lncRNAs and mRNAs in three developmental stages of poplar coma. B. Comparison of the lengths between lncRNAs and mRNAs. E. Comparison of the number of exons between lncRNAs and mRNAs. E. Comparison of the ORF lengths between lncRNAs and mRNAs.

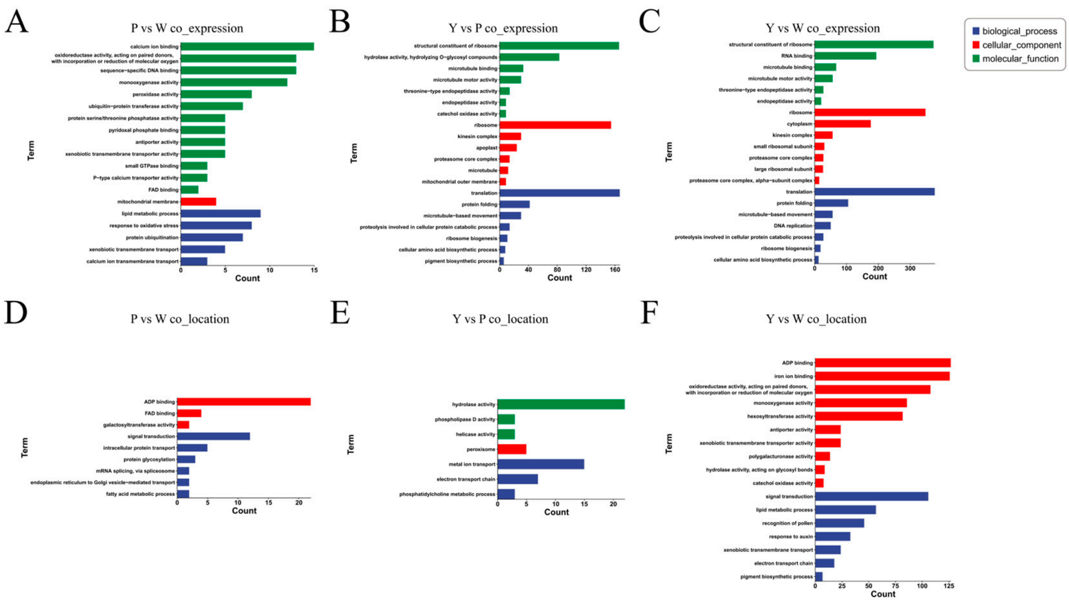

Figure S3. Gene ontology (GO) analysis of differentially expressed lncRNAs(DELs) potential target genes under the molecular function, cellular component and biological processes categories. A. GO analysis of target genes with trans-acting of DELs in PvsW. B. GO analysis of

target genes with trans-acting of DELs in YvsP. C. GO analysis of target genes with trans-acting of DELs in YvsW. D. GO analysis of target genes with cis-acting of DELs in PvsW. E. GO analysis of target genes with cis-acting of DELs in YvsP. F. GO analysis of target genes with cis-acting of DELs in YvsW.

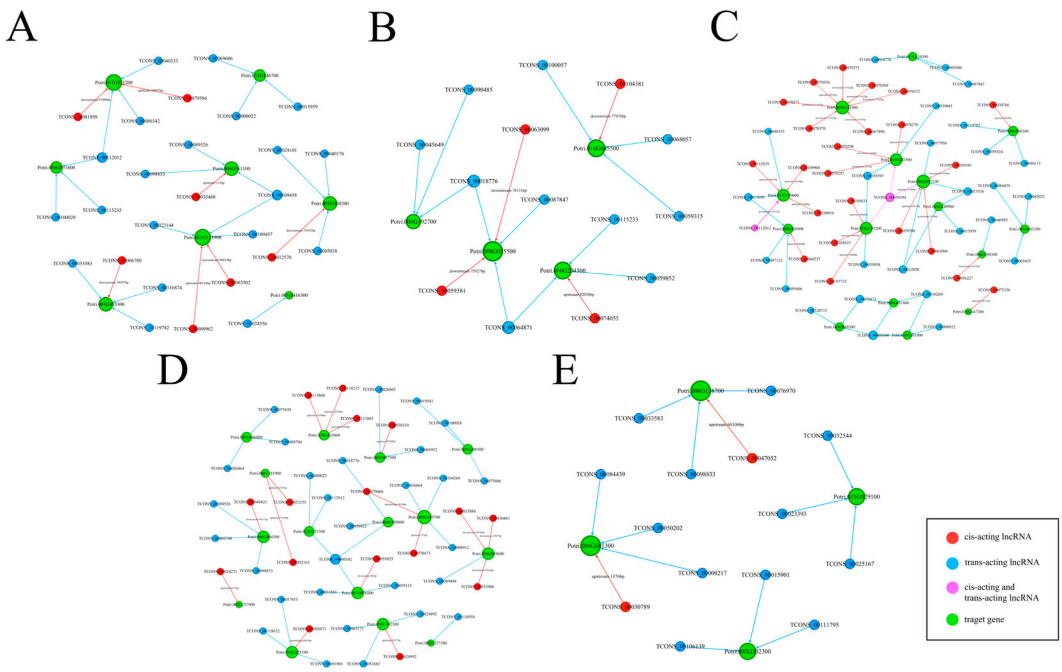

Figure S4. The regulatory relationship between lncRNAs and some genes potentially involved in cell expansion and wall material synthesis during poplar coma development. A. The regulatory relationship between lncRNAs and coding kinesin genes. B. The regulatory relationship between lncRNAs and coding actin genes. C. The regulatory relationship between lncRNAs and coding expansin genes. D. The regulatory relationship between lncRNAs and coding cellulose synthase genes. E. The regulatory relationship between lncRNA and coding sucrose synthase genes. The blue circles represent lncRNAs that regulate target genes through a trans-acting mechanism, The red circles represent lncRNAs that regulate target genes through a cis-acting mechanism, The purple circles represent lncRNAs that simultaneously regulate target genes through both cis- and trans-acting mechanisms, The green circles represent target genes.

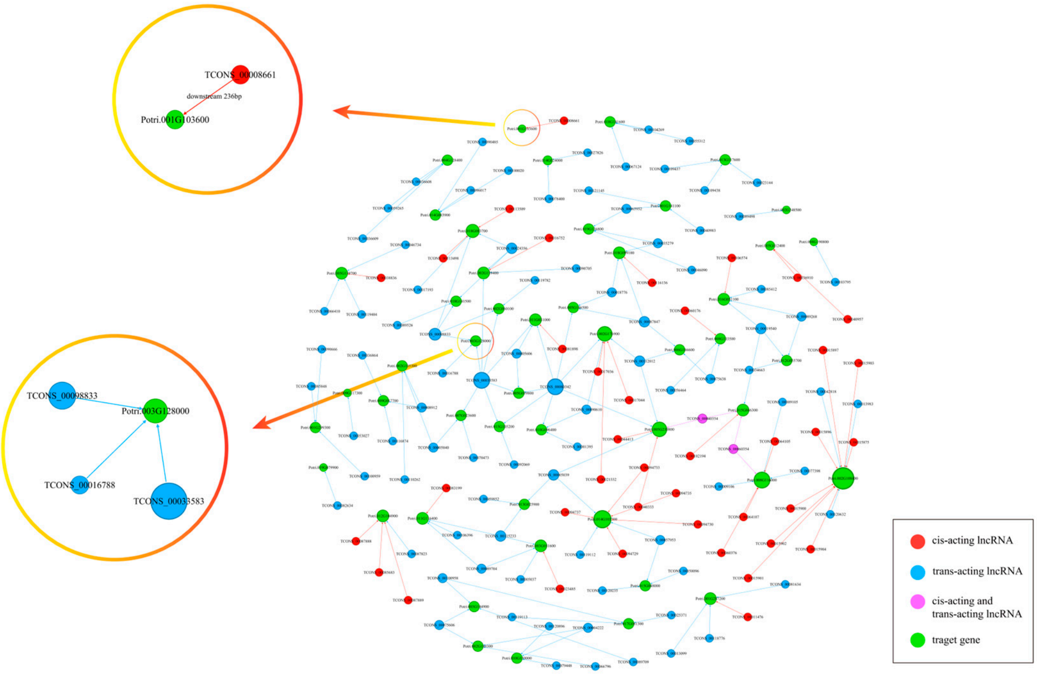

Figure S5. lncRNA-bHLH regulatory networks.

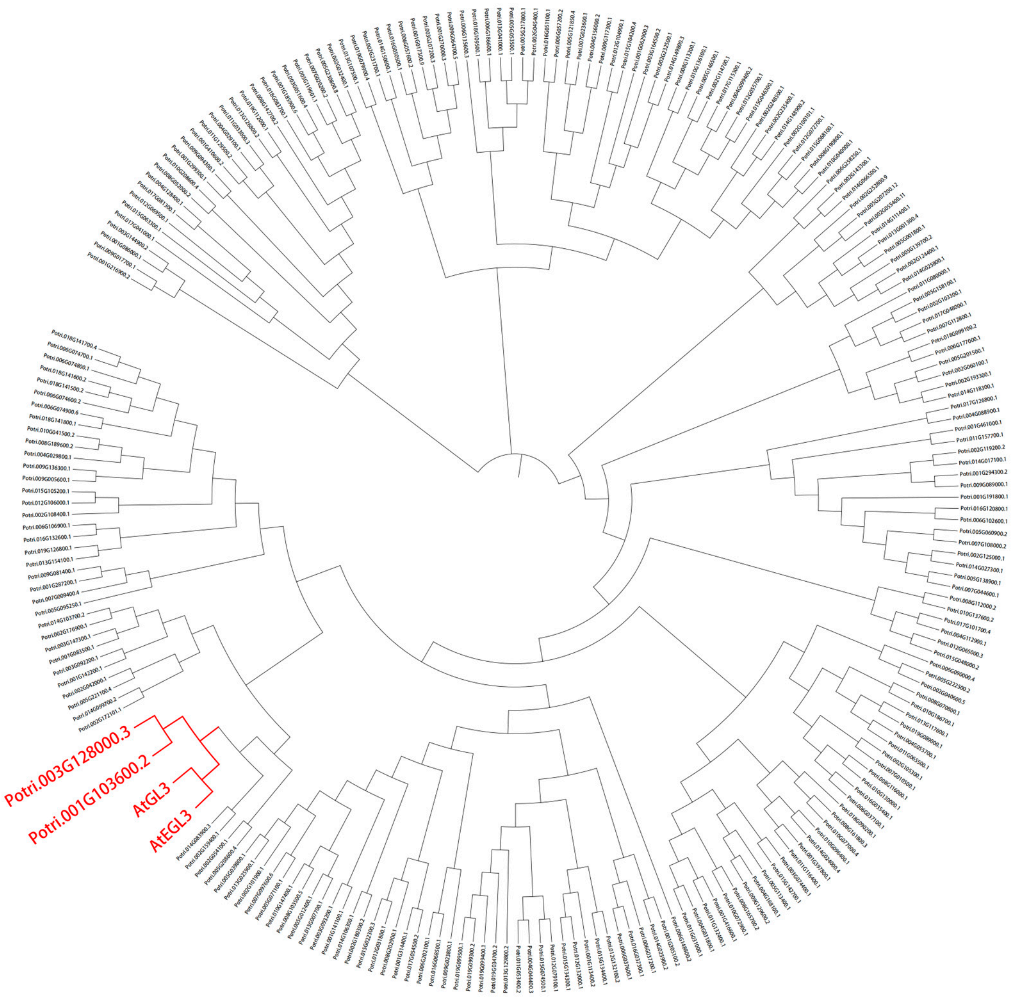

Figure S6. Constructing a phylogenetic tree for the bHLH family using the approximately-maximum-likelihood method.

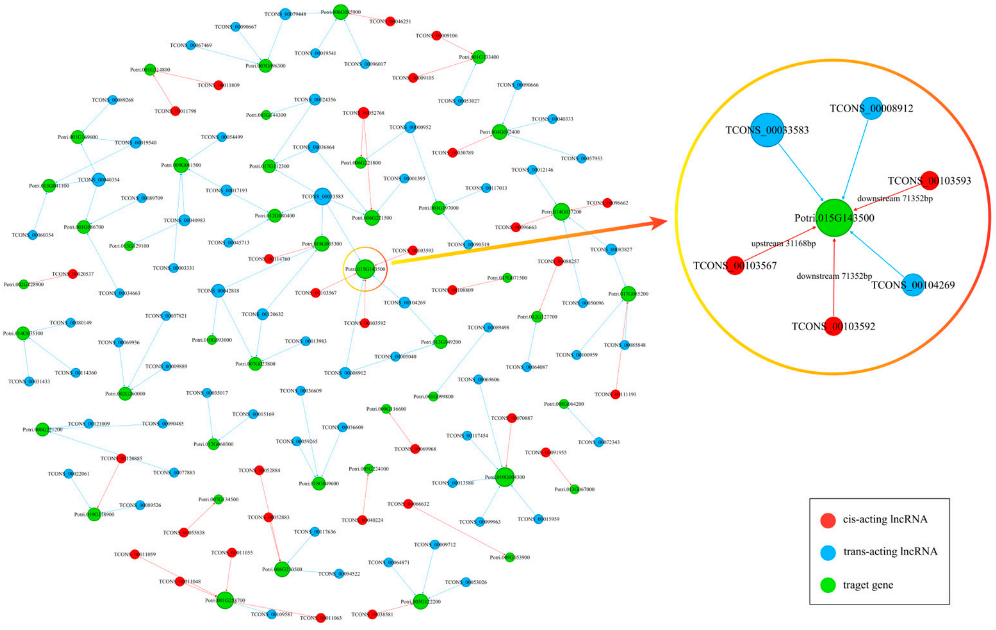

Figure S7. lncRNA-R2R3 MYB regulatory networks.

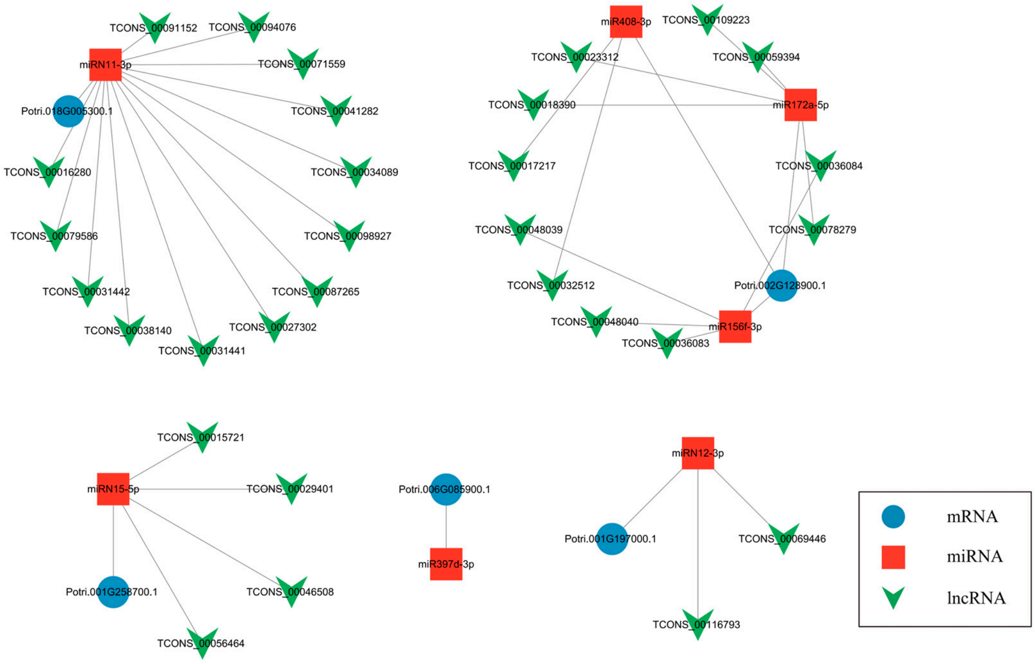

Figure S8. lncRNA-miRNA-R2R3 MYB regulatory networks.

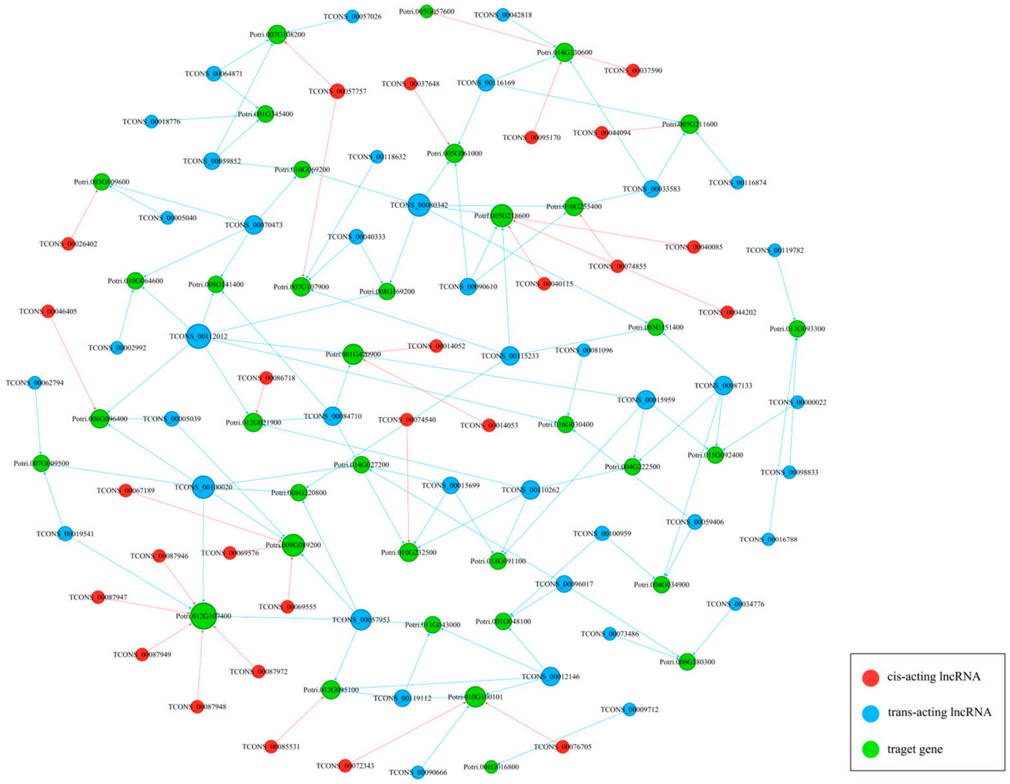

Figure S9. lncRNA-WDR regulatory networks.

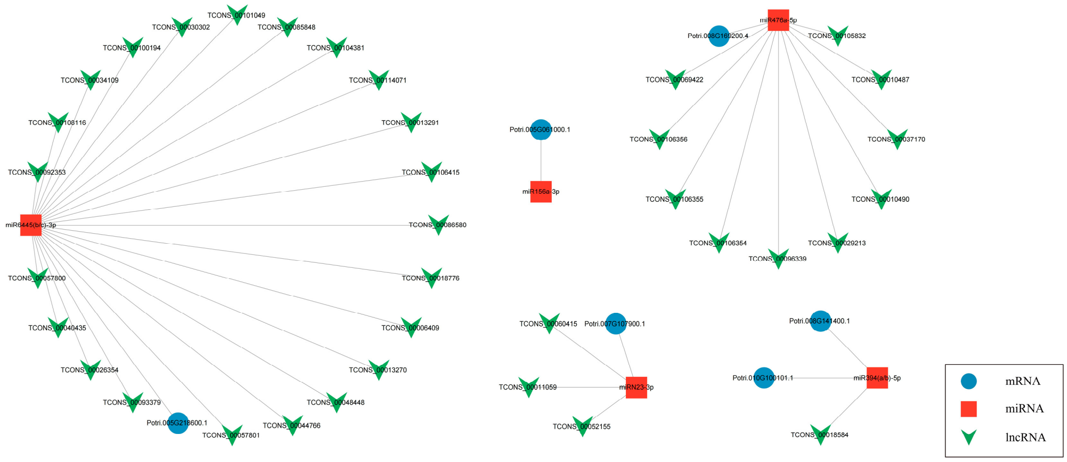

Figure 10. lncRNA-miRNA-WDR regulatory networks.

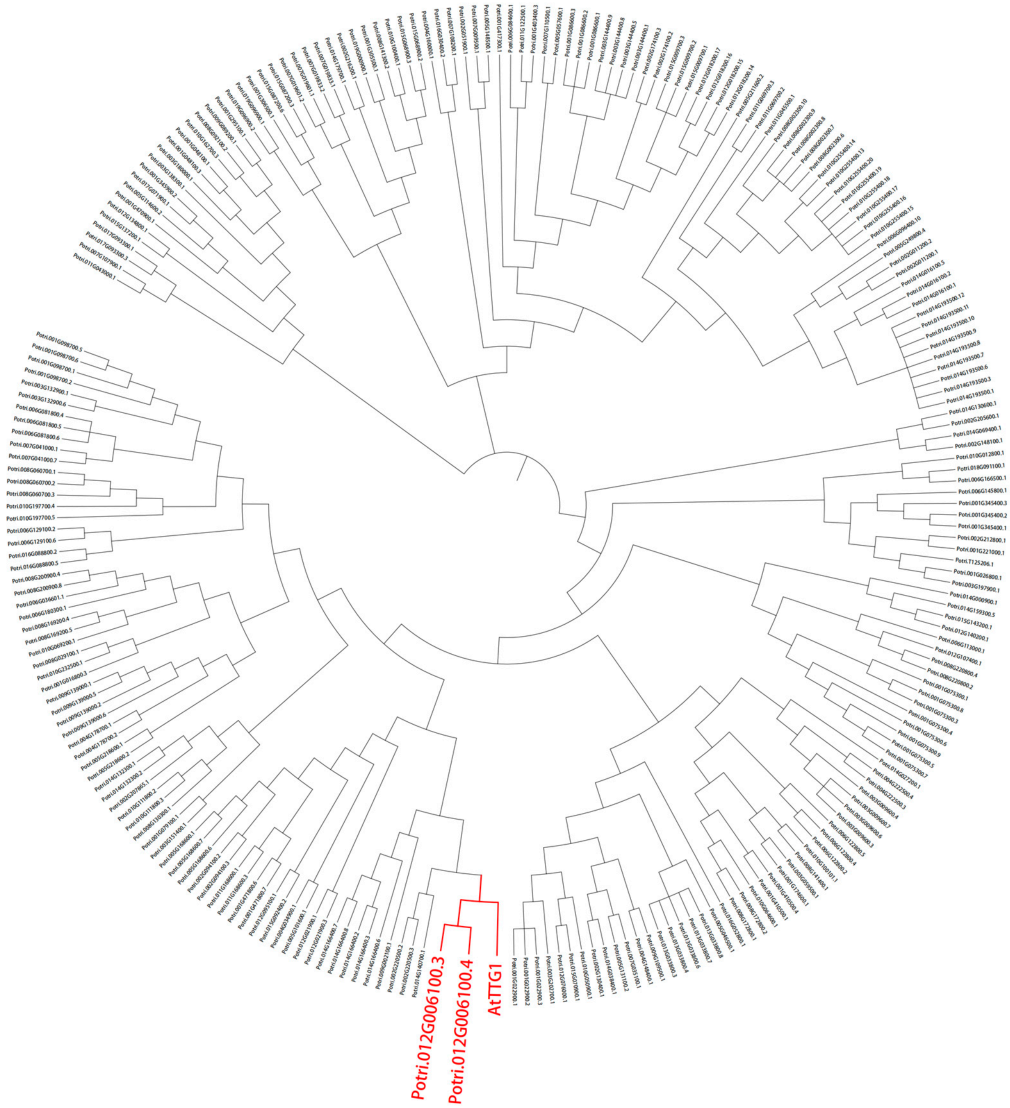

Figure S11. Constructing a phylogenetic tree for the WDR family using the approximately-maximum-likelihood method.
